# Evaluating the efficacy and safety of transcranial pulse stimulation on adolescents with attention deficit hyperactivity disorder: Study protocol of a pilot randomized, double-blind, sham-controlled trial

**DOI:** 10.3389/fneur.2023.1076086

**Published:** 2023-03-28

**Authors:** Teris Cheung, Bolton Chau, Kwan Hin Fong, Joyce Yuen Ting Lam, Herman Lo, Man Ho Li, Albert Martin Man Chim Li, Roland Beisteiner, Sun Lei, Benjamin K. Yee, Calvin Pak Wing Cheng

**Affiliations:** ^1^School of Nursing, The Hong Kong Polytechnic University, Kowloon, Hong Kong SAR, China; ^2^The Mental Health Research Centre, The Hong Kong Polytechnic University, Kowloon, Hong Kong SAR, China; ^3^Department of Rehabilitation Sciences, The Hong Kong Polytechnic University, Kowloon, Hong Kong SAR, China; ^4^Department of Applied Social Sciences, The Hong Kong Polytechnic University, Kowloon, Hong Kong SAR, China; ^5^Department of Psychiatry, The Chinese University of Hong Kong, Shatin, Hong Kong SAR, China; ^6^Department of Paediatrics, The Chinese University of Hong Kong, Shatin, Hong Kong SAR, China; ^7^Department of Neurology, Vienna Medical University, Vienna, Austria; ^8^Department of Biomedical Engineering, The Hong Kong Polytechnic University, Kowloon, Hong Kong SAR, China; ^9^Department of Psychiatry, The University of Hong Kong, Pokfulam, Hong Kong SAR, China

**Keywords:** efficacy, transcranial pulse stimulation, RCT, ADHD, neuromodulation, adolescents

## Abstract

**Background:**

Traditional treatment alone might not effectively control the severity of attention deficit hyperactivity disorder (ADHD) symptoms. Transcranial pulse stimulation (TPS) is a non-invasive brain stimulation (NIBS) technology used on older adults with mild neurocognitive disorders and adults with major depressive disorder. However, there has been no study conducted on young adolescents with ADHD. This will be the first nationwide study evaluating the efficacy and safety of TPS in the treatment of ADHD among young adolescents in Hong Kong.

**Methods:**

This study proposes a double-blinded, randomized, sham-controlled trial including TPS as an intervention group and a sham TPS group. Both groups will be measured at baseline (T1), immediately after the intervention (T2), and at the 1-month (T3) and 3-month follow-ups (T4).

**Recruitment:**

A total of 30 subjects aged between 12 and 17 years, diagnosed with attention deficit hyperactivity disorder (ADHD), will be recruited in this study. All subjects will be computer randomized into either the intervention group or the sham TPS group on a 1:1 ratio.

**Intervention:**

All subjects in each group will have to undertake functional MRI (fMRI) before and after six 30-min TPS sessions, which will be completed in 2 weeks' time.

**Outcomes:**

Baseline measurements and post-TPS evaluation of the ADHD symptoms and executive functions will also be conducted on all participants. The 1- and 3-month follow-up periods will be used to assess the long-term sustainability of the TPS intervention. For statistical analysis, ANOVA with repeated measures will be used to analyze data. Missing data were managed by multiple imputations. The level of significance will be set to *p* < 0.05.

**Significance of the study:**

Results emerging from this study will generate new knowledge to ascertain whether TPS can be used as a top-on treatment for ADHD.

**Clinical trial registration:**

clinicaltrails.gov, identifier: NCT05422274.

## Introduction

Local epidemiological data suggest that attention deficit hyperactivity disorder (AD/HD) affects ~6% of children, with a male preponderance of around 2 boys to 1 girl being affected (1). The prevalence in adults is around 2.5% (2). Clinical features of ADHD are characterized by persistent symptoms of inattention and/or hyperactivity/impulsivity (3) that emerge in childhood (4). These symptoms may persist into adulthood, leading to poor life outcomes, and affecting employment and interpersonal relationships (5). ADHD may affect all aspects of an individual's life and has a negative detrimental impact on family members (6). The neurobiological mechanism of ADHD may be attributed to the dopaminergic imbalance in the forebrain and basal ganglia. The prefrontal cortex, anterior cingulate, insula, amygdala, and cerebellum are also linked to ADHD pathophysiology (7). Typical ADHD treatments include pharmacotherapy, stimulant medications (e.g., methylphenidate; amphetamine), and non-stimulant medications (e.g., atomoxetine) (8) targeting dopaminergic and noradrenergic systems in the frontal cortex and dopaminergic system in the basal ganglia. These medications are effective and safe for the majority of patients; however, 20% of patients do not tolerate these medications or fail to respond (9). Although these medications can significantly improve ADHD symptoms and life outcomes, long-term medication compliance is necessary to sustain the treatment efficacy (10). Drug dosages also need to be individually monitored to minimize adverse effects while maintaining efficacy (8). Whether the long-term risk of taking medications outweighs the benefits in patients with ADHD remains debatable. Although mindfulness-based cognitive therapy (MBCT) has recently been demonstrated as an effective psychosocial intervention (11), the long-term sustainability of the benefits of these psychosocial interventions on ADHD is yet to be confirmed. Pharmacotherapy is not considered as a monotherapy for more than 50% of adult ADHD, (12, 13) and a combination of cognitive behavioral therapy (CBT) and medication produces broader improvements in executive functions in ADHD than CBT alone.

## Neuromodulation and non-invasive brain stimulation (NIBS)

Attempts to design interventions that could directly modulate brain function have received increasing interest with the advent of technology capable of delivering highly focal and tailored modulation of special brain circuit. Non-invasive brain stimulation (NIBS), such as repeated transcranial magnetic stimulation (rTMS) and transcranial direct current stimulation (tDCS), is widely applied to re-balance neural activity at the circuitry level to normalize functions and behavior. Nowadays, these NIBS techniques are being used diagnostically and therapeutically in different types of neurodegenerative diseases (e.g., Alzheimer's disease and Parkinson's disease) (14), pediatric epilepsy (15), neuropsychiatric disorders (e.g., ADHD, major depressive disorder, and substance use disorder) (16), and neurodevelopmental disorders (e.g., autism) (17). A recent systematic review (18) of neurotherapeutics on ADHD presented meta-analytic evidence that EEG-neurofeedback showed small/medium effects compared to non-active controls in randomized controlled trials. Trials evaluating rTMS or tDCS, however, have yielded more mixed outcomes. Nevertheless, rTMS showed inconsistent findings on improving cognition or symptoms in ADHD, while tDCS studies that targeted the dorsolateral prefrontal cortex (DLPFC) showed small effects on cognitive improvements in ADHD. The key findings targeted on specific age groups (e.g., children, adolescents, and adults) of people with ADHD are summarized below (Table 1).

**Table 1:** Findings of non-invasive brain stimulation (NIBS) studies on ADHD.

**References**	***N***	**Age**	**Design**	**Intervention**	**Treatment region**	**Results**
Allenby et al. (4)	37	18–65	Double-blind, sham-controlled randomized controlled trials (RCT)	3 tDCS sessions	Left DLPFC	tDCS improved impulsivity symptoms
Cao et al. (19)	64	6–13	3-armed RCT rTMS (*n* = 20); ATX (*n* = 19); rTMS + ATX (*n* = 21) *ATX = Atomoxetine	6-week rTMS	Right DLPFC	rTMS + ATX group improved significantly in inattention and hyperactivity/impulsiveness at post-treatment (*p* < 0.05). All groups showed improvements in clinical/cognitive measures.
McGough et al. (20)	62	8–12	Double-blind, sham-controlled RCT	4 weeks trigeminal nerve Stimulation (TNS)	Right frontal lobe and frontal midline	Significant reduction of ADHD-RS score (*p* = 0.005) and CGI score on active TNS group (*p* = 0.003) compared to sham TNS group
Soff et al. (21)	15	12–16	Double-blind RCT	5 tDCS	Left DLPFC	Significant reduction of hyperactivity and inattention (*P* < 0.05) but no effect on impulsivity
Paz et al. (22)	22	12–16	Single-blind RCT	20 rTMS	Bilateral DLPFC	No effect on clinical/cognitive outcomes (*p* > 0.05)
Westwood et al. (23)	50	10–18	Double-blind, sham-controlled RCT	15 tDCS	rIFC	No significant improvement in core ADHD symptoms (*p* > 0.05)
Leffa et al. (24)	64	18–60	Double-blind, parallel, sham-controlled RCT	20 tDCs	Anodal-right and cathodal-left prefrontal	Mean inattention score was 18.88 (SD 5.79) in the active tDCS group compared with 23.63 (SD 3.97) in the sham tDCS. Significant treatment by time intervention evaluated by clinician-administered version of the adult ADHD self-report scale (β interaction: −3.18, *P* < 0.001).
Cosmo et al. (25)	60	18–65	Double-blind, sham-controlled RCT	1 tDCS session	Left DLPFC	No significant differences in ADHD symptoms between the tDCS and sham group

In summary, with the exception of trigeminal nerve stimulation (TNS), which has proven a safe and effective intervention for ADHD, other NIBS studies such as EEG-neurofeedback and rTMS/tDCS across different age groups have yielded inconsistent results in ADHD. Almost all NIBS studies primarily focused on left/right/bilateral DLPFC in ADHD. Stimulation targeting the right inferior frontal cortex (rIFC) was shown to be ineffective (26). Since ADHD is increasingly prevalent in Hong Kong, and thus, there is a pressing need to evaluate the efficacy of the latest NIBS technology (such as transcranial pulse stimulation, TPS), not only would such research generate new neuroscientific evidence but would also ascertain whether TPS may be an effective adjunct treatment in ADHD to reduce disease burden and psychiatric morbidity [e.g., mood disorders/anxiety disorders (27), eating disorders, and substance-related disorders (28)] in Hong Kong.

## Mechanisms of TPS

Transcranial pulse stimulation uses repetitive single ultrashort pulses in the ultrasound frequency range to stimulate the brain. With a neuro-navigation device, TPS can target the human brain in a highly focal and precise manner (29). TPS differs from tDCS and rTMS, as these use direct or induced electric current. Using electric currents to stimulate the brain may be limited by the problem of conductivity (30) and failure to reach deep brain regions (31). TPS, however, uses low-intensity focused ultrasound which provides good spatial precision and resolution to non-invasively modulate subcortical areas, despite the problem of skull attenuation (32, 33). By using lower ultrasound frequencies, TPS can stimulate deep cerebral regions, reaching as far as 8 cm into the brain. In other words, TPS can improve skull penetration in the human brain and improve treatment outcomes (29). The biological mechanism of TPS is mechanotransduction. TPS can stimulate vascular growth factors (VEGF) (34, 35) and brain-derived neurotrophic factor (BDNF) (36), improve cerebral blood flow, and promote angiogenesis and nerve regeneration. The ultrashort ultrasound pulse can enhance cell proliferation and differentiation in cultured neural stem cells, and this TPS may play an important role in the repair of brain function in CNS diseases (37).

## Existing research on transcranial pulse stimulation

Ultrasound for the brain is a revolutionary therapeutic treatment approach in patients with neuropsychiatric symptoms (38). Since TPS is a relatively new NIBS technology, only two studies have so far been conducted on the disease population. The first study comprised of 35 Austrian older adults with Alzheimer's disease (AD) who were treated with global brain stimulation in three TPS sessions per week (6,000 pulses each) for 2–4 weeks, with results showing significant improvement in the Consortium to Establish a Registry for Alzheimer's Disease (CERAD) score immediately after intervention and at 1 and 3 months after the intervention. Results from fMRI also showed significantly increased connectivity within the memory network (29). Participants' depressive symptoms were also significantly improved, as measured by the Geriatric Depression Scale (GDS) (*p* = 0.005) and the Beck Depression Inventory (BDI) (*p* < 0.0001) at 1 and 3 months post-stimulation follow-ups compared with the baseline scores (29). The second TPS study was executed by the principal investigator (PI) (Dr. Teris Cheung) of this proposed study. The study evaluated TPS in people with major depressive disorder (MDD) in an open-label pilot randomized controlled trial (RCT) using waitlist controls (WC). A total of 30 subjects (18–51 years) were administered six TPS sessions over 2 weeks (total TPS pulse: 1,800–2,400, 2.5–3.0 Hz). Results showed significant improvement in the depression severity in the verum TPS group compared to WC (*p* = 0.02), and the effect size was very large (Cohen's *d* = −0.9) (39). However, both Beisteiner et al. (29) and Cheung et al.'s (39) studies were uncontrolled studies or open-label RCTs without a sham control group. Placebo effects have to be considered when interpreting results. Since then, there has been no further attempt to use TPS on neurodevelopmental disorders in children or young adolescents in Hong Kong and nationwide. The impetus of our proposed research is to fill this research gap, which could be critical for the management of ADHD.

### Treatment region

In this trial, we will target the left dorsal lateral prefrontal cortex (DLPFC). The selected brain region is based on previous tDCS research that left and right DLPFCs (40) are primarily the brain treatment regions for ADHD and that stimulation of left DLPFC, specifically, can effectively improve inattention and hyperactivity (4, 21).

## Objectives

The aim of this study is (1) to evaluate the efficacy and safety of TPS on young adolescents (12–17 years) with ADHD in Hong Kong; (2) to examine the association between TPS and ADHD core symptom severity, executive function, inattention, hyperactivity, impulsivity, and oppositional defiance; and (3) to examine the brain functional connectivity changes immediately after the 2-week TPS treatment *via* neuroimaging.

## Hypotheses

### Primary hypothesis

Participants in the verum TPS group will have a 30% reduction in the Swanson, Nolan, and Pelham Rating Scale (SNAP IV score) (i.e., attention deficit, hyperactivity impulse, and oppositional defiance) after 2 weeks of TPS treatment compared with the sham TPS group and be maintained at the 1- and 3-month follow-ups.

### Secondary hypotheses

1. Participants in the verum TPS group or the sham TPS group will have < 5% somatic discomfort during the 2-week TPS intervention on young adolescents with ADHD.
2. Participants in the verum TPS group will have a 30% improvement in ADHD symptoms and behavior compared with the sham TPS group after 2 weeks of TPS treatment and be maintained at the 1- and 3-month follow-ups.
3. Participants in the verum TPS group will have a 30% improvement in executive function after 2 weeks of TPS treatment compared with the sham TPS group and be maintained at the 1- and 3-month follow-ups.
4. Participants in the verum TPS group will have a 30% improvement in both attention deficit and reduction in hyperactivity and impulsivity after 2 weeks of TPS treatment compared with the sham TPS group, and be maintained at the 1- and 3-month follow-ups.
5. Participants in the verum TPS group will have more brain connectivity changes after 2 weeks of TPS compared with the sham TPS group and be maintained at the 1- and 3-month follow-ups.

## Trial design

**Figure 1:**
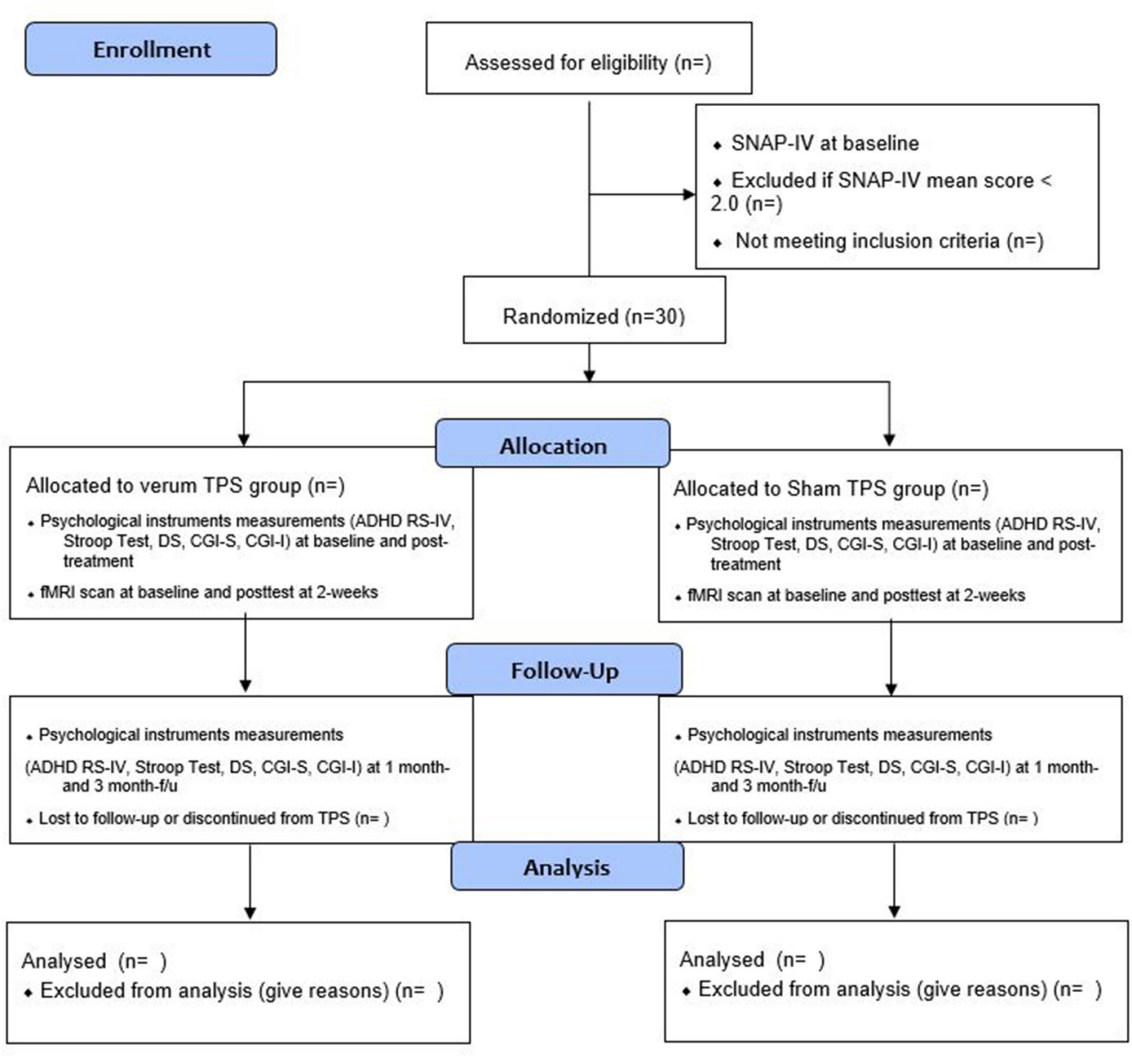
Flow diagram for subjects' enrollment, randomization, allocation, and follow-up.

This proposed study is a two-armed, randomized, double-blind, sham-controlled trial evaluating the efficacy and safety of a 2-week TPS treatment on young adolescents with ADHD. The trial design complies with the Consolidated Standards of Reporting Trials (CONSORT) statement (41). Participants will be randomly allocated into the verum TPS group or sham TPS group. All the participants' parents will be informed about the randomization procedures and that they have a 50% chance of receiving the verum TPS or the sham TPS. This study will be conducted in accordance with the Declaration of Helsinki (42). Both groups will be measured at baseline (T1), immediately after the 2-week intervention (T2), and at 1- and 3-month follow-ups (T3) (43) (Figure 1).

## Methods

### Subjects

Participants will be recruited *via* a mass email invitation attached with a QR code poster that will be delivered *via* members of the Hong Kong Association for ADHD, CUHK, and HKU. A QR code flier will also be flagged up in communal areas on campus. The recruitment period will span 2 months. All participants will require parental written consent for TPS treatment and neuroimaging. Both participants and their parents will be informed that this study involves random allocation into either a sham or treatment group, and the possible side effects of TPS will be clearly stated in the information sheet.

### Inclusion criteria

The inclusion criteria are as follows: (1) those who have a confirmed diagnosis of ADHD according to the 5th edition of the Diagnostic and Statistical Manual of Mental Disorders (DSM-5) of the American Psychiatric Association; (2) those with Chinese ethnicity, aged 12–17 years, with no co-morbidity of other mental disorders (e.g., intellectual disability disorders) or organic brain diseases that affect cognitive functions; (3) those who have no severe systemic diseases including heart, liver, lung, and kidney diseases; (4) those who have an IQ of >80 by Stanford–Binet Intelligence Scale, 5th Edition (SB-5); and (5) those who have written consent from parents.

### Exclusion criteria

The exclusion criteria are as follows: (1) SNAP IV score < 1; (2) those who had not taken ADHD medication in the previous 2–4 weeks; (3) those who had been treated with TMS/rTMS/tDCS or electroconvulsive therapy in the previous 12 months; (4) those who had taken monoamine oxidase inhibitors in the previous 14 days; (5) those who have a history of epilepsy, brain trauma, brain surgery/brain tumor, brain aneurysm, or other concomitant unstable major medical conditions like hemophilia or other blood clotting disorders or thrombosis; (6) those who have significant communicative impairments; (7) those who have metal implants in the brain treatment region or artificial cardiac pacemaker *in situ*; (8) those who had had corticosteroid treatment within the previous 6 weeks before the first TPS treatment; and (9) those who have a history of micro-cavernomas.

### Sample size

To the best of our knowledge, there is no interventional study evaluating the efficacy of TPS on ADHD. Based on our previous open-label pilot RCT (39) evaluating TPS in adults with major depressive disorder that showed a large effect size (d = 0.91), we hypothesize a large effect of TPS in this study. We used G^*^power version 3.1.9.4 to calculate the target sample size. With a statistical power of 95% and a statistical significance level of 0.05 to detect a medium between-groups effect size (d) of 0.91 with four measurement time points, each group will require 15 subjects. A total sample of 30 is required in this trial. The attrition rate in our pilot MDD trial was 0%. We expect that the attrition rate in this ADHD trial would be < 5%. Subjects dropping out of the 2-week intervention period will be replaced by another enrolled subject in this pilot study.

### Screening and self-administered questionnaire

Participants' parents will complete a QR code online application form soliciting sociodemographic information [age, gender, educational background, monthly family household income, living circumstances, school year, participant's psychiatric history, and duration of ADHD diagnosis (in years/months)], age of diagnosis, duration of taking prescribed medications (in years/months), current drugs and dosages, and family history of psychiatric disorder.

Eligible subjects will then fill in the screening tool (The Swanson, Nolan, and Pelham Rating Scale (SNAP IV), and those with a SNAP IV mean score of >2 will be recruited. Subjects' medical history, treatment regime, and developmental history will be obtained by direct inquiry with subjects' parents either by Zoom interview or Facetime before neuroimaging and TPS treatment. Both participants and parents will be interviewed by the PI and the research personnel. Parents need to hold a valid medical certificate of his/her child's ADHD diagnosis and a prescribed formulation sheet during the online interview. Any parent who fails to show this proof will not be invited to participate in the trial.

### Randomization, allocation, and masking

All consenting participants will be listed in alphabetical order according to their surnames, and each participant will be assigned a unique identifier. An independent statistician (Dr. Li Man Ho) will use a computer-generated list of random numbers (www.random.org) to ensure the concealment of randomization. Randomization will be conducted by an independent statistician off-site using a stochastic minimization program to balance the gender, age, and SNAP-IV scores of the participants. Block randomization with blocks of 10 (total: 3 blocks) will be used to allocate treatment groups. Participants from each block will be randomly assigned to the verum TPS groups or the sham TPS groups on a 1:1 ratio. To avoid information flow, participants/parents and research associates will be blinded to the group allocation to minimize potential contamination of the effects of TPS or subject bias. The interventionist will not be involved in data collection or pre- and post-TPS measurements. Outcome measurements will be conducted by a research associate who is not involved in the group allocation. Participants and their parents will be asked to guess the grouping (verum TPS vs. sham TPS) in the last TPS session to determine the probability of guessing the group allocation correctly in the subject blinding (44).

### Interventions

TPS intervention will be performed at the Integrative Health Clinic, at the Hong Kong Polytechnic University. A licensed mental health practitioner (PI: Dr. Teris Cheung) will deliver the intervention.

### TPS procedures

**Figure 2:**
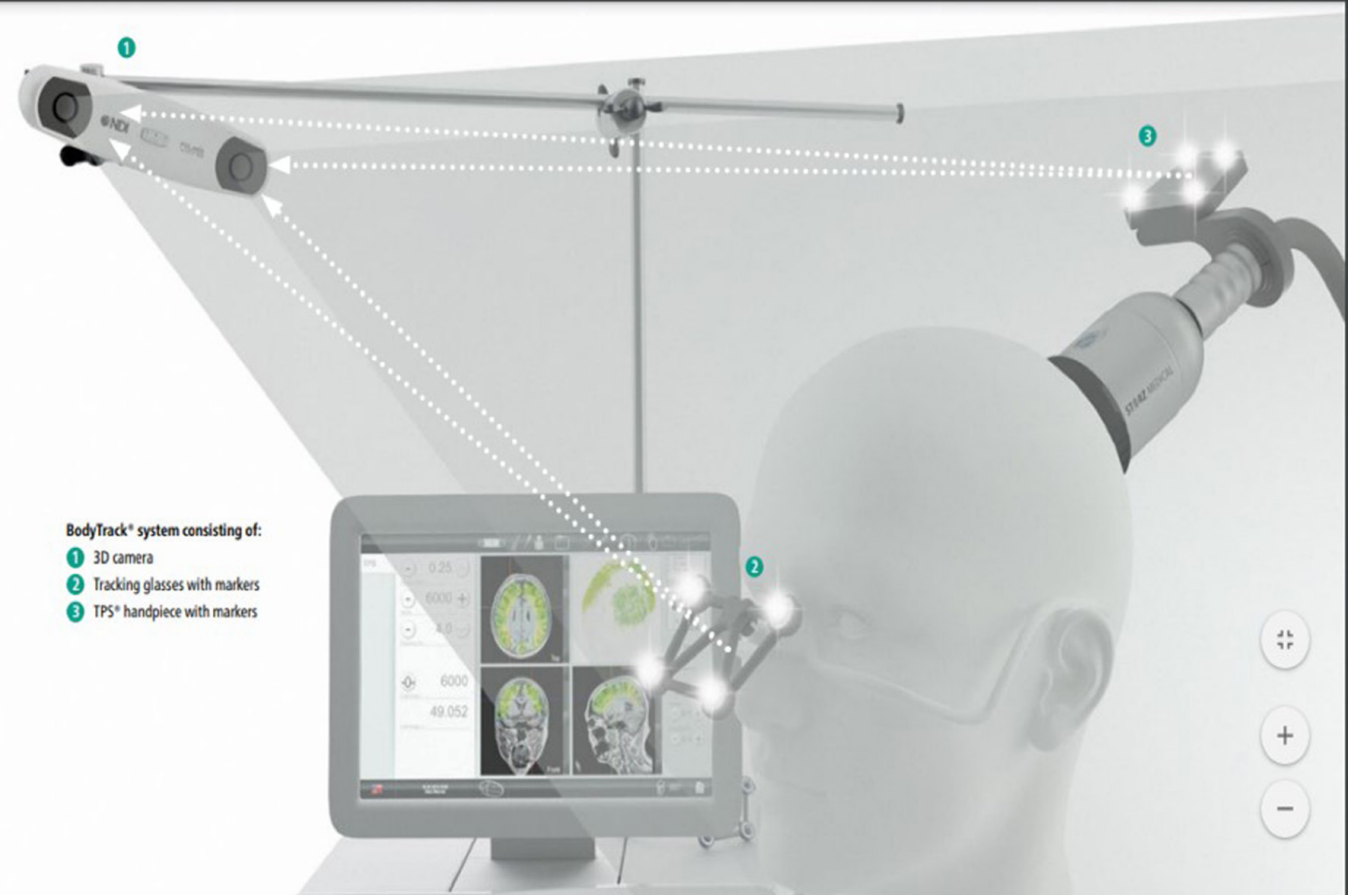
Transcranial pulse stimulation (TPS) system.

**Figure 3:**
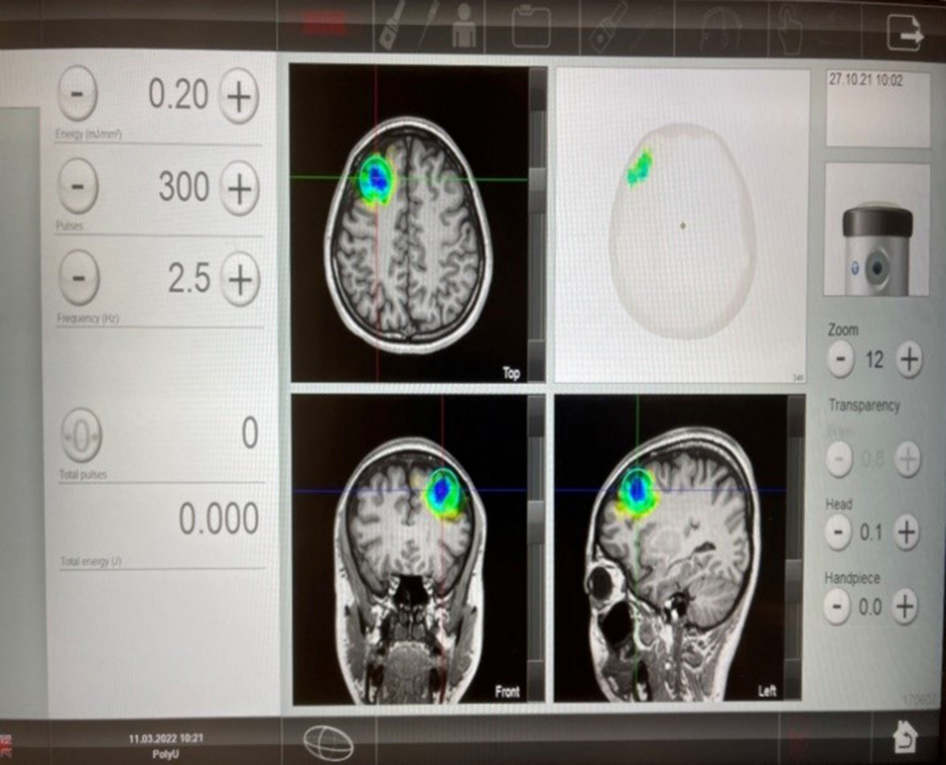
Transcranial pulse stimulation (TPS) (STORZ MEDICAL) post-intervention images.

**Figure 4:**
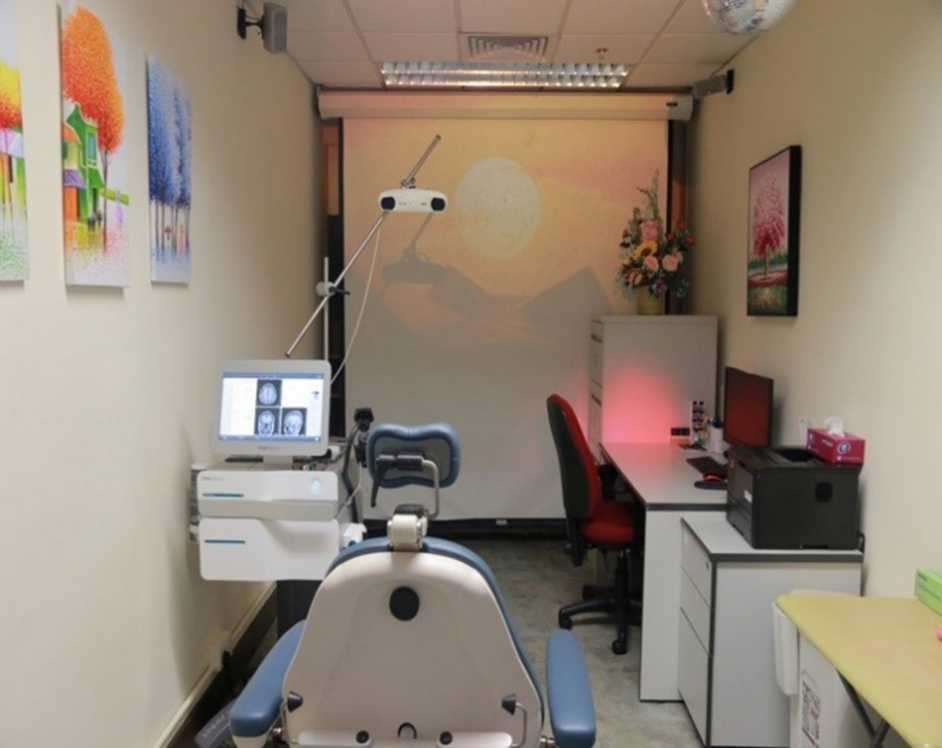
Transcranial pulse stimulation system and TPS treatment venue in IHC/PolyU.

The TPS system consists of a mobile single transducer and an infrared camera system for MR-based neuro-navigation (NEUROLITH, Storz Medical AG, Tägerwilen, Switzerland). TPS generates single ultrashort (3 μs) ultrasound pulses with typical energy levels of 0.2–0.25 mJ/mm^2^ and pulse frequencies of 4–5 Hz (pulses per second). During the TPS session, participants will be sitting in a comfortable electronic chair in the treatment venue. Participants will wear a BodyTrack system consisting of a 3D camera, tracking glasses with markers, and a TPS handpiece with markers (Figure 2). This BodyTrack system ensures that the participant's head matches with his/her fMRI T1 images previously taken in UBSN so that each TPS pulse applied can be visualized and documented in real-time. Real-time tracking of the handpiece position enables automatic visualization of the treated brain region. The energy applied will be highlighted in green (Figure 3). The interventionist will use the variable stand-offs at the handpiece for depth regulation and manual movement of the handpiece over the skull with real-time visualization of participants' fMRI brain images. The whole treatment session will be recorded for *post-hoc* evaluation of the individual intracerebral pulse localizations (Figure 4).

### TPS Intervention dose

In this proposed study, we will deliver 800 pulses to the subject's left DLPFC in each session (total: 4,800 pulses). All participants (in both active and sham TPS groups) will receive six 30-min TPS sessions over 2 weeks (i.e., 3 sessions/week, on alternate days, total treatment time: 3 h) using energy levels of 0.25 mJ/mm^2^ and a frequency of 4 Hz. We believe that a 2-week TPS intervention will be sufficient enough to test the efficacy of TPS on ADHD (29, 39). Participants will be followed up immediately after stimulation at 2 weeks, 1 month, and 12 weeks (Figure 1). We believe that a post-treatment follow-up of up to 3 months is sufficient to evaluate the sustainability of TPS on ADHD (29, 39).

### Sham TPS

Participants will be given an identical TPS intervention dose, but the silicone oil used in the verum TPS group will be replaced by an air-filled cushion in the handpiece. Participants will also hear sounds and stimuli similar to the verum TPS group.

### Fidelity

To ensure the fidelity of the intervention, the project team will ascertain whether the interventions will be delivered as intended. The interventionist (PI) obtained a Ph.D. in social sciences (HKU) and is a UK- and HK-licensed mental health professional with more than 10 years of clinical experience in mental health and neuroscience. The research associate will issue WhatsApp message reminders (e.g., TPS intervention schedule, fMRI scan appointments, and f/u appointment slips) to parents and will report subjects' progress, and indicate adverse effects and adherence throughout the trial period.

### Safety, adverse effects, and risk indicators of TPS

TPS uses very low energy for brain stimulation, thus TPS intervention should not cause any serious adverse effects such as intracranial bleeding, edema, or other intracranial pathology, as confirmed in previous studies (29, 39). Although this TPS system has obtained clinical certification (CE) indicating that it is a safe intervention, we will prepare a checklist stating all the potential adverse effects associated with TPS administration (29) and monitor the subject's tolerability and adverse events in each session throughout the trial period. In the pilot RCT on MDD (39), a few subjects reported transient headaches (< 2 h) (4%) but none required pain analgesics. Nonetheless, all subjects will be covered by master trial insurance in this study.

### Ethical and data security considerations

Participants' data in both groups will be stored in two separate datasets with an identifier linking these data. Both sets of data will be encrypted using TrueCrypt (http://www.truecrypt.org). The data from the baseline and 12-week follow-up will be linked according to personal data. All precautions in data protection will be taken, as suggested by TrueCrypt. To prevent leakage of personal data, only the PI will have access to the personal dataset. Written consent will be obtained from all participants and both parents. An information sheet containing the purpose of this trial and the potential risks and benefits of its procedures for undertaking MRI scans in UBSN/PolyU and TPS will be provided to all parents. Participants' parents will be informed of their children's anonymity; withdrawal or non-compliance will not result in any consequences.

## Outcome evaluation (primary and secondary outcomes)

### Baseline assessment

#### Demographic data

The subjects' basic demographic data, including age, gender, body mass index, years of education, birth history, number of siblings, monthly household income, and first-degree family members' history of ADHD (yes/no), will be collected upon study entry. Details of the subjects' psychiatric history, including the age of diagnosis and any developmental delays or serious injury on any bodily parts, or serious physical illness(es), will also be recorded at the baseline assessment.

### Primary outcome

#### Attention deficit, hyperactivity impulse, and oppositional defiance

The Swanson, Nolan, and Pelham Rating Scale (SNAP IV) will be used to measure participants' attention deficit, hyperactivity impulse, and oppositional defiance. SNAP IV consists of 26 items summarized into three factors: attention deficit, hyperactivity impulse, and oppositional defiance. Parents, based on their general impressions of their children, rate the severity of symptoms on a Likert scale (0–3). A mean score of <1 indicates “normal” or “remission”; a mean score of 1 is defined as the demarcation between attention deficit and hyperactivity-impulsivity; a mean score of >2 indicates “abnormal.” SNAP-IV is a reliable and valid scale used in RCTs (45) and has good psychometric properties that can be used for the Chinese population (46).

### Secondary outcomes

#### Clinical Global Impression

The Clinical Global Impression (CGI)—severity and improvement scale (CGI-S and CGI-I) is generally used to assess illness severity and global improvement. CGI-S is a 7-point clinician rating scale based on observed and reported symptoms, behavior, and function in the past 7 days. CGI-I is a 7-point scale to assess whether the patient's ADHD condition has improved or worsened compared to the baseline. CGI-S and CGI-I will be used to supplement each other (47). These scales had been used in a double-blinded placebo-controlled RCT (48).

#### Executive function

The Stroop test is a neuropsychological test commonly used to assess the inhibition control component of executive function and test the subject's ability to inhibit cognitive interference that occurs when the processing of the target stimulus feature is impeded by the simultaneous processing of a second stimulus attribute (49).

#### ADHD symptoms and behavior

The ADHD Rating Scale–IV (ADHD RS-IV) (50, 51) is a widely used ADHD scale comprising 18 items. The participant's parent rates the frequency of each symptom on the scale. Each item is scored on a 4-point Likert scale of 0–3 (0: never or rarely; 1: sometimes; 2: often; and 3: very often). The nine odd items evaluate attention deficits, composing the inattention subscale (or IA); the nine even items evaluate hyperactivity-impulsivity, composing the hyperactivity-impulsivity (or HI) subscale; the total score is the sum of all the scores on the 18 items. The ADHD RS-IV is a reliable and valid scale that can be used for the Chinese population (52).

### Neuroimaging

Participants will receive pre- and post-treatment MRI scans (total: two MRI scans) to measure any changes in structural and functional connectivity changes in the brain. Structural MRI, DTI, and rs-fMRI will be performed using a 3T scanner at the UBSN (ZB216), The Hong Kong Polytechnic University. The subjects will be closely monitored by the research assistant and the radiographer during scanning. The whole scan will last around 30 min including preparation. Structural MRI scans including T1 sequences will be used for assessing regional volume differences across the whole brain. High-resolution sagittal 3D T1-weighted (SPGR/MPRAGE) images of 1 × 1 × 1 mm will be acquired with a repetition time (TR) = 1,820 ms, echo time (TE) = 3.75 ms, inversion time (TI) = 1,100 ms, and flip angle = 70°. DTI sequence will be conducted using single-shot spin-echo echo-planar imaging, with diffusion-sensitizing gradients applied along 16 non-collinear directions with diffusion weighting factor b = 1,000 s/mm^2^, plus two b = 0 images. The imaging parameters will be TR/TE = 1,200/82 ms, matrix size = 128 × 128, the field of view (FOV) = 240 mm, slice thickness = 3 mm with no intersection gap, number of excitations = 2, and number of slices = 67. Finally, resting-state fMRI of 150 T2-weighted gradient echo planar imaging (EPI) will be acquired with TR = 2 s and TE = 32 ms; 32 slices, with a resolution of 3 × 3 × 4 mm, during which subjects will view a fixation cross (“+”) passively at the center of the screen. Images processing and analysis will be performed using software packages including FSL (http://fsl.fmrib.ox.ac.uk/fsl/fslwiki/). Total brain and total gray and white matter volumes will be extracted from the T1 structural scan. Gray matter and white matter tissue maps will be segmented and compared for regional tissue density differences using voxel-based morphometry (VBM) (53). Structural connectivity will be assessed by fractional anisotropy (FA) maps extracted from DTI imaging.

For functional connectivity, all resting state-fMRI (rs-fMRI) volumes will be pre-processed, with motion correction and slice timing correction, then linearly registered to the Montreal Neurological Institute (MNI) standard space. A data-driven approach will be used for the analysis of rs-fMRI data. Independent component analysis will be done with Multivariate Exploratory Linear Decomposition in FSL. A set of independent components will be identified as the common resting-state functional networks. The global and local efficiency, modularity, and hubs will be computed using the Brain Connectivity Toolbox (https://sites.google.com/site/bctnet/). A dual regression approach will be used to investigate between-group differences in the individual functional networks. The significance threshold of the voxel-wise differences will be set at *p* < 0.05 (family-wise error corrected).

### Statistical analyses

All statistical analyses will be performed using the statistical software R for Windows (R version 4.1.0). Means and standard deviations (SD) for the continuous variables will be presented, while numbers and percentages for the categorical variables will be shown. A *p*-value of < 0.05 is considered statistically significant. Sociodemographic differences between the TPS group and the sham TPS group will be analyzed using the Chi-square test and *t*-test. If there are significant differences between sociodemographic factors, covariates will be considered confounding variables in the analyses. The normality of the primary outcome (SNAP-IV) scores will be tested by the Shapiro–Wilk test for each combination of factor levels (group and time). *T*-test will be used to test the baseline difference. A linear mixed model will be used to test the group (between-subject factor), time (within-subject factor), and group × time interaction effects of the SNAP-IV score between the TPS group and the sham TPS group. *Post-hoc* comparisons between groups and time points will be conducted using a *t*-test with Bonferroni correction. The normality of the secondary outcome scores will be tested by the Shapiro–Wilk test for each time point. For normally distributed outcomes, a linear mixed model will be used to determine whether the outcome scores are significantly different between the pre- and post-tests. For outcome scores that deviate grossly from normality, a non-parametric Friedman test will be used to test the mean difference. A Cohen's *d* effect size for each outcome will be calculated, where *d* = 0.2, 0.5, and 0.8 correspond to small, medium, and large effect sizes (54). Missing data will be managed by multiple imputations (55). For the neurological rs-fMRI data, a longitudinal voxel-based morphometry (VBM) will be used to examine whether TPS produces local changes in gray matter. Specifically, relative local increases and decreases between pre- and post-intervention scans will be compared within and between study groups. The diffusion MR data will be analyzed using the diffusion tensor model (56). Two standard diffusion indices will then be obtained: the apparent diffusion coefficient and the fractional anisotropy. Pre- and post-treatment DTI scans will permit the assessment of changes in axial diffusivity, with lower values being interpreted as the structural enhancement of white matter.

## Discussion

This study is the first RCT to evaluate the efficacy and safety of TPS in patients with ADHD nationwide. Findings that emerge from this project will have a significant impact on patients/caregivers and the community at large. Findings will inform health policymakers on whether TPS could be used as an adjunct treatment in the clinical setting in psychiatry—given the fact that both medications and psychotherapy require long-term input to sustain the therapeutic effects of ADHD. As such, this inevitably increases health costs, the caregiving burden, and the global disease burden. If this project can prove that TPS is effective in the treatment of patients with ADHD, it could instill hope in the patients' families and reduce their psychological burden to a large extent that ADHD is curable and treatable by TPS. This would be a breakthrough in neuroscience research specific for adolescents with special education needs (SEN) in Hong Kong.

## Trial status

This trial was registered with clinicaltrails.gov on 10 August 2022 (protocol version). Recruitment commenced from 1 June 2022 to 30 Sept 2022.

## Ethics statement

The studies involving human participants were reviewed and approved by Human Subjects Ethics Sub-Committee, The Hong Kong Polytechnic University. Written informed consent to participate in this study was provided by the participants' legal guardian/next of kin.

## Author contributions

TC, CPWC, and RB conceived the study. TC, CPWC, and BKY designed the study. JYTL and KHF executed the study and administered the TPS. JYTL arranged neuroimaging logistics. JYTL and KHF assisted in the administration of TPS and baseline and post-TPS measurements and also in data entry and administration of baseline and post-TPS measurements. MHL assisted in the randomization process. MHL, JYTL, and HKF assisted in statistical analysis. RB, BC, CPWC, and BKY offered expert advice in fMRI data analysis. AMMCL, TC, CPWC, RB, MHL, SL, and HL assisted in manuscript writing. All authors contributed to the article and approved the submitted version.
